# Peritonitis due to Moraxella Osloensis: An Emerging Pathogen

**DOI:** 10.1155/2018/4968371

**Published:** 2018-12-20

**Authors:** Sreedhar Adapa, Purva Gumaste, Venu Madhav Konala, Nikhil Agrawal, Amarinder Singh Garcha, Hemant Dhingra

**Affiliations:** ^1^Attending Nephrologist, The Nephrology Group, Fresno, CA, USA; ^2^Attending Physician, Kaweah Delta Medical Center, Visalia, CA, USA; ^3^Medical Director and Medical Oncologist, Ashland Bellefonte Cancer Center, Ashland, KY, USA; ^4^Fellow, Nephrology, Beth Israel Deaconess Medical Center, Harvard Medical School, Boston, MA, USA; ^5^Program Director, Internal Medicine Residency, St Agnes Medical Center, Fresno, CA, USA

## Abstract

Peritonitis is a very serious complication encountered in patients undergoing peritoneal dialysis and healthcare providers involved in the management should be very vigilant. Gram-positive organisms are the frequent cause of peritonitis compared to gram-negative organisms. There has been recognition of peritonitis caused by uncommon organisms because of improved microbiological detection techniques. We report a case of peritonitis caused by Moraxella osloensis (M. osloensis), which is an unusual cause of infections in humans. A 68-year-old male, who has been on peritoneal dialysis for 2 years, presented with abdominal pain and cloudy effluent. Peritoneal fluid analysis was consistent with peritonitis and peritoneal fluid culture grew gram-negative bacteria. M. osloensis was identified by 16 S PCR phenotypic and sequencing techniques. Patient responded well to the treatment, with intraperitoneal cephalosporin, and repeat peritoneal fluid culture yielded no growth. M. osloensis rarely causes infection in humans and responds well to treatment, as reported in literature.

## 1. Introduction

M. osloensis is a gram-negative coccobacillus, which is a commensal of human skin and mucosa. It rarely causes infection in humans. Infections from these bacteria have been reported in immunocompromised patients [1]. These bacteria are very susceptible for penicillins, cephalosporins, and aminoglycosides. To date only two cases of peritonitis from M. osloensis have been reported in literature [2, 3].

## 2. Case Report

A 68-year-old male with history of end stage renal disease presented with abdominal pain and cloudy effluent for one-day duration. Patient has been on automated peritoneal dialysis for 2 years and never had an episode of peritonitis. Patient admitted working in his garden one day prior to the presentation. Other medical problems include hypertension, diabetes, and anemia of chronic disease. Patient was afebrile and vital signs were stable. Physical examination revealed diffuse abdominal tenderness and no drainage from exit site. No tenderness was elicited along the tunnel of peritoneal dialysis catheter. Peritoneal dialysis effluent showed elevated WBC with cell count 1991 cells/ul (with 94% neutrophils). Gram stain revealed few WBC and no organisms were seen. Effluent grew gram-negative rods both in aerobic and anaerobic cultures. Confirmation of M. osloensis was made by 16S PCR phenotypic and sequencing technique. Susceptibilities were not performed on this organism by the reference laboratory, as no Clinical & Laboratory Standards Institute (CLSI) guidelines are available. Patient was empirically treated with intraperitoneal ceftazidime 1 gram daily for 3 weeks. Post treatment peritoneal dialysis effluent was clear with WBC count <20 cells/ul and repeat fluid culture was negative.

## 3. Discussion

Peritonitis is a well-recognized complication in patients undergoing peritoneal dialysis and is often the reason for change in dialysis modality from peritoneal dialysis to hemodialysis. Most common organisms causing peritonitis are gram-positive bacteria from skin flora accounting for more than 60% of cases [4]. Peritonitis from gram-negative bacteria is less frequent and fungal peritonitis is rare. Peritonitis from Moraxella catarrhalis has been reported frequently, but only two cases of peritonitis by Moraxella osloensis have been reported to date [2, 3].

Moraxella is a genus of aerobic, gram-negative, nonmotile, oxidase positive coccobacilli [5]. They were initially included in genus Neisseria and later transferred to genus Branhamella and finally got the status of their own [5]. Moraxella are normal commensals of skin and mucosa. These are unusual pathogens in humans except M. catarrhalis, which causes upper and lower respiratory tract infections. The other medically important species are M. atlantae, M. lacunata, M. nonliquefaciens, and M. osloensis [5].

M. osloensis has been reclassified as a separate species in 1967 [6] and prior to that has been grouped along with M. nonliquefaciens. The bacterium has been isolated from multiple environmental sources like sinks, laundry [7], anesthetic agents [8], and slug parasite nematode [9]. Our patient had a history of gardening, the day before the presentation with peritonitis, which could have led to the touch contamination, as the bacterium is present in slug parasite nematode. Fewer than 40 cases of infections by M. osloensis have been reported in literature causing bacteremia, endocarditis, meningitis, osteomyelitis, and pneumonia [1, 10–15].

M. osloensis is not promptly identifiable and pleomorphic appearance on gram stain adds to the diagnostic dilemma. New technologies are playing a critical role in identifying M. osloensis, which was underdiagnosed previously. RNA gene sequence analysis is the primary method of diagnosing the bacterium and accurate diagnosis is of utmost importance [16].

There are no established guidelines on the treatment of M. osloensis because of sparse cases of infection caused by the bacteria in literature. Penicillins, cephalosporins, aminoglycosides, fluoroquinolones, and carbapenems are reported to be effective in treating M. osloensis [1]. Removal of peritoneal dialysis catheter is not required, as in our patient.

## 4. Conclusion

Early recognition and institution of effective therapy will prevent complications and change of modality in peritonitis. M. osloensis is a very rare cause of peritonitis; prompt diagnosis and accurate treatment are the key factors in the management. Its ubiquitous presence, including slugs and laundry, can be a potential source of infection like in our patient.
